# Hypertension and diabetes screening uptake in adults aged 40–70 in Indonesia: a knowledge, attitudes, and practices study

**DOI:** 10.1186/s44263-025-00157-7

**Published:** 2025-05-13

**Authors:** Maja E. Marcus, Anna Reuter, Lisa Rogge, Farah Diba, Marthoenis, Sebastian Vollmer

**Affiliations:** 1 Department of Economics, University of Goettingen, Göttingen, Germany; 2 Brigham and Women’s Hospital, Boston, USA; 3 Harvard Medical School, Boston, USA; 4 Charité – Universitätsmedizin Berlin, Berlin, Germany; 5 Heidelberg Institute of Global Health, University of Heidelberg, Heidelberg, Germany; 6 Bundesinstitut Für Bevölkerungsforschung, Wiesbaden, Germany; 7 Leibniz University of Hannover, Hannover, Germany; 8 FAU Erlangen-Nürnberg, Nuremberg, Germany; 9 Universitas Syiah Kuala, Banda Aceh, Indonesia; 10 Department of Psychiatry and Mental Health Nursing, Universitas Syiah Kuala, Banda Aceh, Indonesia

**Keywords:** Cardiovascular diseases, Diabetes, Prevention, Health knowledge, Health care seeking

## Abstract

**Background:**

Diabetes and hypertension are major global health crises, yet Indonesia is lagging behind in achieving care outcomes compared to other middle-income countries. We examined barriers to screening uptake, a key care entry point, in 40–70-year-old adults in Aceh, Indonesia.

**Methods:**

We assessed individual-level data on diabetes and hypertension screenings in Banda Aceh and Aceh Besar in 2019. Using two-stage random sampling, we collected survey data on 2080 adults that are indicated for, but had not undergone diabetes screening as per World Health Organization’s Package of Essential Noncommunicable Disease Intervention guidelines. Using this, we adjusted the data for complex survey design to describe (1) the share of respondents with screening indication and presence of risk factors; (2) disease-related knowledge, attitude, and practices, as well as (3) estimate associations of screening with socioeconomic characteristics, knowledge, and attitudes using multivariable linear and logistic regression.

**Results:**

We found that while respondents were aware of diabetes and hypertension, a majority lacked knowledge about leading risk factors, the conditions’ potentially asymptomatic nature, and screening needs. About 41% of respondents never had any blood pressure or glucose check, the primary reason reported being not feeling ill. Blood glucose checks were rarely conducted. We found rural location and lower education to be associated with lower disease-related knowledge, and lower wealth with lower knowledge and screening uptake.

**Conclusions:**

Barriers to screening uptake in Aceh, Indonesia, include misconceptions around hypertension and diabetes, provider-specific challenges especially around the provision of glucose testing, and socioeconomic gradients.

**Supplementary Information:**

The online version contains supplementary material available at 10.1186/s44263-025-00157-7.

## Background

Low- and middle-income countries (LMIC) across the globe are facing an increasing burden of cardiovascular diseases (CVD) [1]. In Indonesia, 38% of all deaths were caused by CVD in 2019—one of the highest CVD death rates in Southeast Asia [2]. Responding to this health burden, Indonesia aims to improve screening and management services for diabetes and hypertension, two leading risk factors and therefore critical targets in the prevention of CVD. In the last decade, the country established opportunistic screening services at every community health center (Puskesmas) and community-based screening programs at the village-level (Posbindu) free of cost [3, 4]. Within the Posbindu program, health staff from the community health centers visits each participating village once a month and offers basic health check-ups, including CVD screening, and health information for free.

Despite these efforts, diabetes and hypertension screening remain heavily underutilized [5]. While the World Health Organization’s Package of Essential Noncommunicable Disease Intervention (WHO PEN) and Indonesia’s Posbindu guidelines specify that anyone over the age of 40 should undergo regular CVD screening, around one-third of Indonesians aged 45 and older have never had a blood pressure test and around 70% never had a blood glucose test [5–7]. The underutilization of CVD screening holds especially true in our study region, Aceh province, where diabetes and hypertension rates are above the national average and screening rates below [5].

At the provider-level, mixed-method studies on the Posbindu program reported both supply-side and demand-side barriers to screening uptake [8, 9]. These pertain to difficulties in reaching and engaging patients, low screening awareness, and implementation challenges. Studies at the individual-level remain rare despite their significance for comprehensively capturing and quantifying demand-side barriers to diabetes and hypertension screening uptake. One study assessed knowledge, attitude, and practices (KAP) for hypertension between Posbindu participants and non-participants and found that hypertension knowledge, but not practice, is higher among Posbindu participants [10]. In other low- and middle-income contexts, individual-level KAP studies towards diabetes and hypertension showed that higher socioeconomic status was associated with regular blood glucose screening as well as that most respondents knew at least some risk factors and complications of diabetes and hypertension [11–17].

We complement this literature with a KAP study on the barriers of hypertension and diabetes screening uptake in 40–70-year-old adults in Aceh, Indonesia. In this, we contribute to the previous literature in four ways: (i) we specifically examine a sample of individuals from the general population who are indicated for diabetes and hypertension screening as per WHO PEN and national guidelines; (ii) we examine their KAP in a setting in which common barriers to screening uptake are largely reduced through free, local screening offers; (iii) we examine socioeconomic gradients in KAP; and (iv) we compare the KAP of two major CVD risk factors, namely hypertension and diabetes, using a harmonized set of questions.

A Bahasa Indonesia translation of the abstract is provided in Additional file 1. 

## Methods

### Data sources

We collected individual-level data on knowledge, attitudes, and practices via in-person interviews in Aceh, Indonesia, in November and December 2019. The target population were individuals indicated for CVD screening as per WHO PEN and national guidelines, but who have not undergone screening accordingly [6, 7]. As such, we included individuals between 40 and 70 years old that were not screened for diabetes during the previous year (as per self-reports). We excluded individuals who reported that they were already diagnosed for hypertension or diabetes, were in continued care, or did not have access to a phone of their own or of an appointed contact person. Phone access was required for the purpose of a field experiment on screening uptake that followed the data collection [18]. The sample was randomly selected in two stages: first, a set of 152 villages was drawn from a stratified list of all villages from the provincial capital district Banda Aceh and its surrounding district Aceh Besar. Within the villages, households were selected through a standardized random walk scheme: A village subdivision was randomly selected as starting point. Enumerators counted houses to their left, turning left at crossings, and turning around at the end of a road. Households were selected based on a counting rule dependent on the village size to enable a wide coverage of the village area. Within the households, one eligible member was randomly selected for an interview. A full list of the enumerator instructions can be found in a prior publication on the data [19]. One-third of all contacted households had a household member that met our inclusion criteria. For further details on power calculations, sampling, and excluded households, refer to prior publications on the data [18, 19]. In total, 2100 individuals were interviewed, but only 2080 were included in the analysis as it became clear during the interviews that, in contrast to their statements during the eligibility check, 20 individuals already received hypertension or diabetes diagnoses and thus did not meet the inclusion criteria.

### Variable definitions and measurements

#### Screening indication

For both diabetes and hypertension, we defined two binary variables describing whether an individual is recommended to be screened for each respective health condition based on the WHO PEN guidelines (see Additional file 1: Table S1). Specifically, the first binary variable (“(A) Age of 40 + years”) describes screening indication based on being 40 years or older—which by definition of our sample inclusion criteria applies to all survey respondents. The second binary variable (“(B) Additional CVD risk factors”) describes a composite measure of screening indication of whether the respondent reported at least one additional risk factor put forth by the PEN guidelines. For diabetes, these risk factors are as follows: (i) physical inactivity, (ii) having a household member with diabetes, (iii) having a history of CVD, and (iv) having high cholesterol. For hypertension, these risk factors are: (i) having a history of CVD, (ii) smoking, and (iii) having a household member with a history of heart attack, stroke, or diabetes.

In addition to these composite indication variables, we defined a binary variable for each of the aforementioned risk factors. Finally, we defined two more binary CVD risk factor variables of relevance in the Indonesian context: (i) the consumption of sugar-sweetened beverages (SSB) and (ii) having inflammatory arthritis [20, 21].

All measures were based on self-reported survey items. Physical inactivity and tobacco consumption were measured closely following the survey instrument of the World Health Organization’s STEPwise approach to non-communicable disease (NCD) risk factor surveillance (WHO STEPS). We deviated from the WHO STEPS instrument in our measure of physical activity by not distinguishing between moderate and vigorous physical activity during work or leisure. Instead, we measured any physical activities as “activities that make you breathe harder than normal and may include carrying loads, diffing, plowing, aerobics, bicycling, or mopping the floor” [22]. Respondents reported whether they engaged in any physical activity within the last 7 days, and if so, on how many days they engaged in physical activities and how long they engaged in it on a usual day of activity (less than 30 minutes, 30 minutes to less than 2 hours, 2 hours to less than 4 hours, 4 hours or more). The midpoints of the duration intervals were multiplied with the number of days to yield an upper bound of physical activity in the last week. The same procedure was applied to assess walking in the past week. Physical inactivity was then defined as engaging in less than 150 minutes of continuous physical activity or walking within the past 7 days, based on the WHO recommendations for physical activity [23]. Tobacco consumption was defined as currently smoking any tobacco products or consuming smokeless tobacco products. Consumption of SSB was defined as drinking at least one SSB, including sugared hot drinks or syrup, on a regular day [24].

#### Diabetes and hypertension knowledge and attitudes

We measured hypertension and diabetes knowledge and attitudes via a set of self-reported survey items, closely following Fottrell et al. [15]. Knowledge items were measured by asking unaided questions on complications, risk factors, means of control, and screening targets for diabetes and hypertension. Attitudes were measured on a four-point Likert scale as agreement to statements derived from Becker’s health belief model [25]. Examples are the perceived likelihood to be affected by hypertension or diabetes, the detectability and treatability of both conditions, and fear of being affected (refer to Fig. 2 for a list of all indicators and Additional file 1: Table S2 for the full list of statements). As extreme responses (“strongly agree” or “strongly disagree”) were rare, answers were aggregated into two categories (“agree/strongly agree” and “disagree/strongly disagree”).

#### Screening practices

We measured screening activity using self-reported survey items on whether and where the respondent was screened. We defined *ever screened* as the respondent ever having had either a blood pressure or blood sugar test. Conditional on ever being screened, we defined *hypertension screening* as having had a blood pressure test during the last screening visit, and *diabetes screening* as having had a blood sugar test during the last screening visit. We defined screening locality as whether the last blood pressure or blood sugar testing occurred at (i) the village-based screening program Posbindu (*Posbindu*); (ii) the Puskesmas (*PKM*); or (iii) a private doctor or midwife (*Private Doctor*). As a supplementary analysis, we reported reasons why respondents did not go for screening.

### Statistical analyses

We conducted descriptive analyses on all above-described outcomes within the dimensions of (i) screening indication, (ii) diabetes and hypertension knowledge and beliefs, and (iii) screening practices. We further used a multivariable linear regression model to analyze the factors associated with diabetes and hypertension knowledge and beliefs, and a multivariable logistic regression model to analyze the factors associated with screening uptake. To this end, diabetes and hypertension knowledge and belief items were collapsed into factors (for each condition separately) using factor analyses. Factors with an eigenvalue of at least 1 were retained and rotated (oblimin oblique rotating). Higher index values indicate higher knowledge in the respective dimension. We assessed the correlates of screening uptake for each of the outcomes *ever screened*, *hypertension screening*, and *diabetes screening* separately, and the correlates of the screening locality for each of the outcomes *Posbindu*, Puskesmas (*PKM*), and *Private Doctor* separately. We included indicators for being over 50, being female, and living in Banda Aceh (*urban*), as well as education and wealth categories as explanatory variables in all regressions. As additional analyses, we re-run the regressions including the knowledge and belief indices obtained from the factor analyses (results shown in the Additional file 1). The regression and factor analyses were adjusted for sampling design, specifying villages as the primary sampling unit and urban or rural residency as stratification, to obtain correct variance estimations. For the reporting of means and shares of respondents, missing values were dropped on an analysis-by-analysis basis, for the regression analyses, a complete-case analysis was applied. All analyses were conducted using Stata version 16 [26].

## Results

### Screening indication

The 2080 survey respondents were on average 50 years old, 64% were female, and more than two-thirds had more than primary education (see Additional file 1: Table S3). Due to the stratification of our sample, almost half lived in Banda Aceh (urban area). A comparison of these sociodemographic characteristics to individuals of the same target group (40–70 years old, with access to a mobile phone), who were surveyed for the Indonesian National Socioeconomic Survey (SUSENAS), showed similar patterns (see Additional file 1: Table S3) [27]. An overview of missing data in all variables of interest is shown in the Additional file 1 (see Additional file 1: Table S4).

**Fig. 1:**
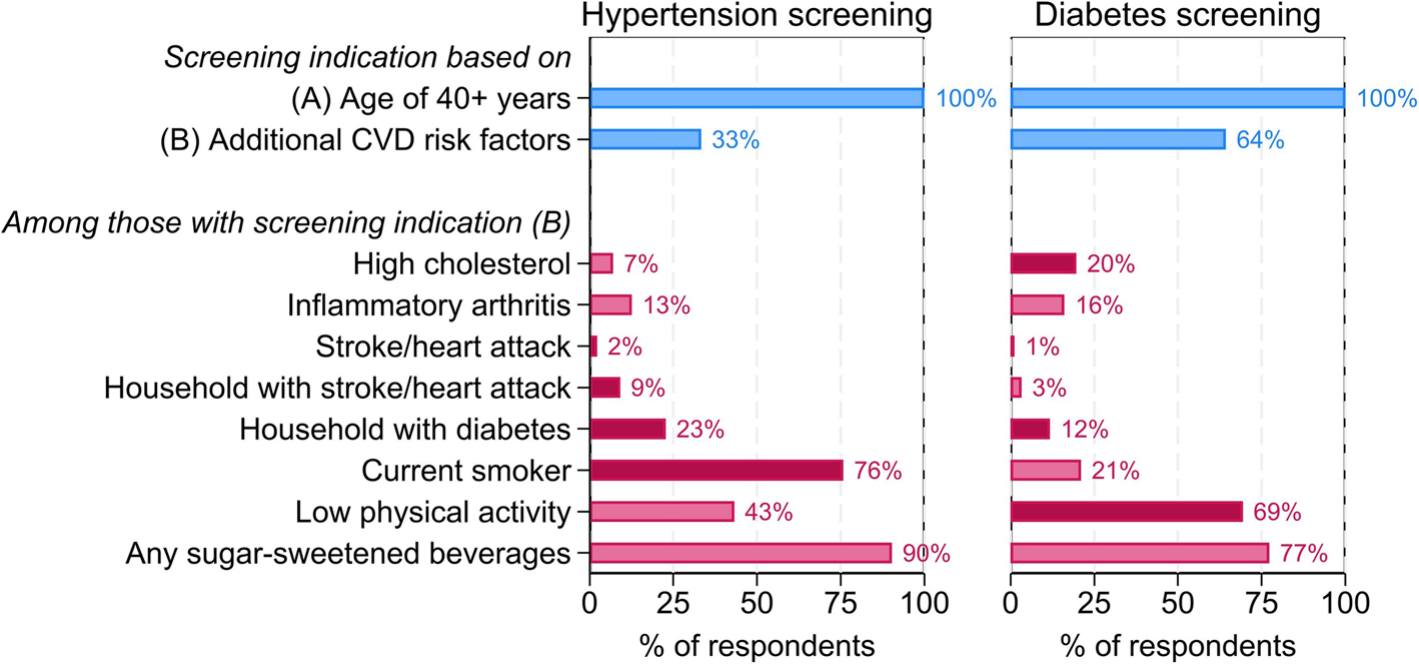
Occurrence of risk factors. Note: Share of respondents with screening indication and the respective risk factors in percent. Bars in dark color represent the risk factors which indicate screening need for blood pressure respectively blood glucose according to WHO PEN guidelines

As depicted in Fig. 1, the sample population was characterized with several risk factors indicating blood pressure and blood glucose screening. Given our age-based inclusion criterion (40 years or older), all respondents had a screening indication due to their age. For this reason, we highlighted those who with a screening indication due to other risk factors in addition to their age. Among those with additional risk factors indicating hypertension screening (33% of the sample), smoking was the most prevalent screening indicator (76%), followed by preconditions of other household members, namely diabetes (23%) and heart attack or stroke (9%), and own experience of heart attack or stroke (2%). Moreover, 7% had cholesterol and 13% inflammatory arthritis, 43% had low levels of physical activity, and 90% consumed at least one SSB on a regular day. Among those with additional risk factors indicating diabetes screening (64% of the sample), 20% had high cholesterol, 12% a household member with diabetes, and 69% had low levels of physical activity. Also, 16% had inflammatory arthritis, 1% previously experienced a heart attack or stroke, 3% had a household member with a previous heart attack or stroke, 21% smoked, and 77% consumed at least one SSB on a regular day. Further details on the respondent’s and other household members’ preconditions are shown in Additional file 1: Fig S1.

In general, behavioral risk factors were highly prevalent in the sample. As depicted in Additional file 1: Fig S2, smoking risk was almost exclusively driven by men, of which almost all smoked daily, and consumed on average 10–14 cigarettes per day. Data from the national health statistics suggest that this high prevalence of smoking among men is neither age- nor region-specific (Additional file 1: Fig S3). Over 80% of the respondents regularly consumed at least one SSB a day, of which the most commonly consumed drink was sugared coffee (Additional file 1: Fig S4). About one-fifth of the respondents were at increased risk due to low physical activity. As depicted in Additional file 1: Fig S5, there was a large divide among respondents, with nearly 40% reporting neither walking nor conducting any moderate or vigorous physical activity on any day of the past week, and about one-third reporting daily activities. Walking for more than 30 minutes a day was seldomly reported, but nearly half of the respondents engaged in physical activities for over 30 minutes, and 25% in activities lasting more than 4 hours (Additional file 1: Fig S6).

### Diabetes and hypertension knowledge and beliefs

**Fig. 2:**
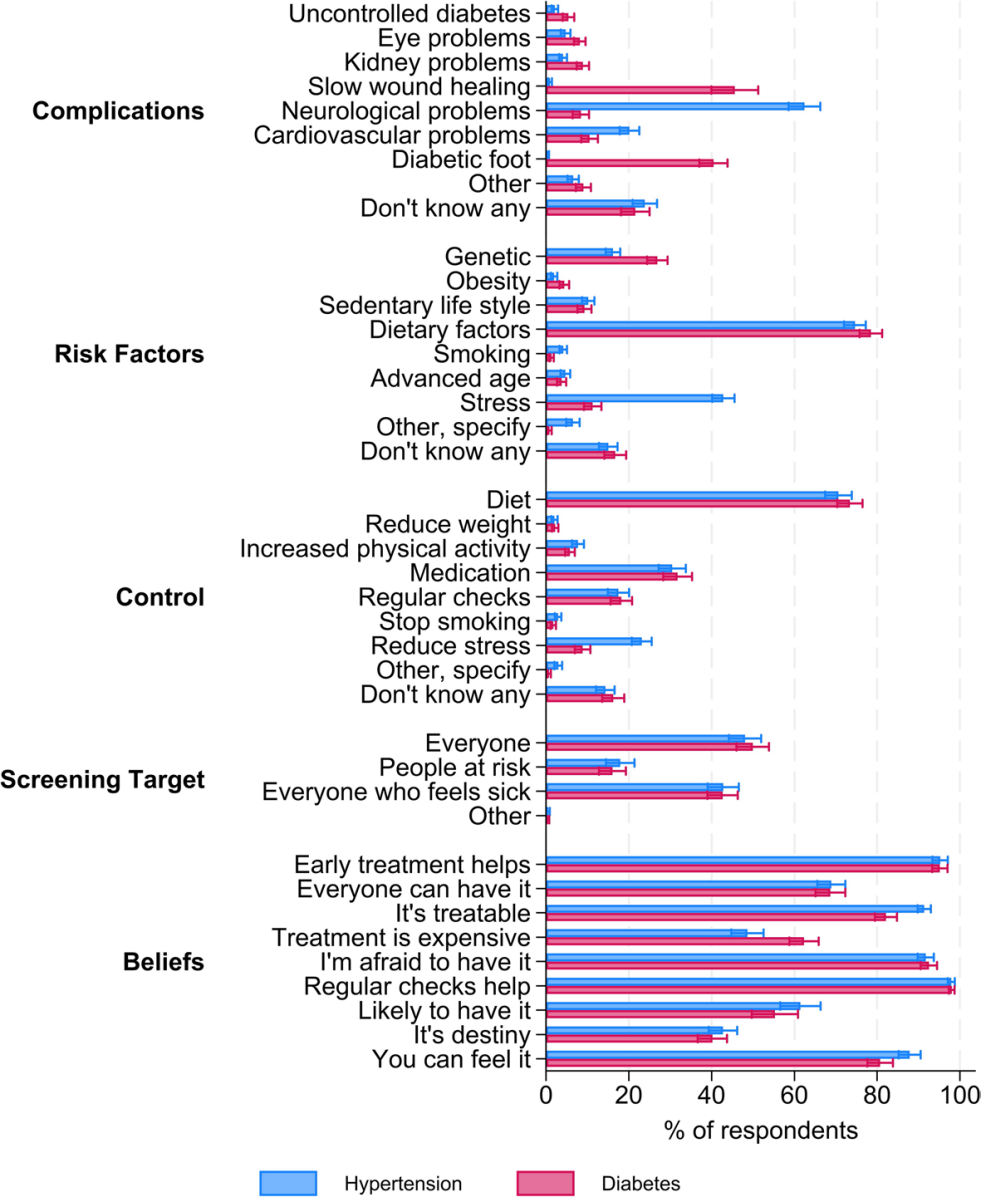
Knowledge and beliefs on hypertension and diabetes. Note: The questions on complications, risk factors, ways to control, and screening target were unaided recall questions. The questions on beliefs could be answered on a four-point Likert scale; displayed is the share of respondents answering with “agree” or “strongly agree.” Share of respondents who reported the respective knowledge or belief with 95% confidence interval

We found moderate levels of diabetes and hypertension risk factor knowledge among our respondents. Approximately three out of four respondents—75% (95% confidence intervals (CI) 72–77%) and 78% (CI 76–81%) respectively—mentioned diet as a risk factor for hypertension and diabetes, followed by stress for hypertension (43%, CI 40–46%; diabetes 11%, CI 9–13%) (Fig. 2). Genetic factors were mentioned by 16% (CI 14–18%) in the case of hypertension and 27% (CI 24–29%) in the case of diabetes. Other major risk factors—such as physical inactivity, obesity, tobacco consumption, or advanced age—were recalled by less than 10% of our sample. Individuals with a family member with CVD risk had higher odds to name genetics as a risk factor for hypertension and diabetes, but there was no significant correlation of other individual dispositions with knowing the respective risk factor once controlling for age, gender, education, and rural/urban location (Additional file 1: Table S5). About one out of six respondents could not name any risk factor for hypertension (15%, CI 13–17%) or diabetes (17%, CI 14–19%).

The most commonly known measure to control diabetes and hypertension was diet (hypertension 71%, CI 67–74%; diabetes 73%, CI 70–76%), followed by medication (hypertension 30%, CI 27–34%; diabetes 32%, CI 28–35%). In the case of hypertension, this was followed by 23% (CI 21–26%) of respondents mentioning stress reduction and 18% (CI 15–20%) mentioning regular checks. For diabetes, 18% mentioned regular checks (CI 16–21%) and 9% (CI 7–11%) stress reduction. None of the control factors could be named by 14% (CI 12–17%) of respondents for hypertension and 16% (CI 14–19%) for diabetes.

When asked for complications arising from hypertension, 62% (CI 59–66%) named a neurological complication (e.g., stroke), 20% (CI 18–23%) named cardiovascular factors (such as a heart attack), and 24% (CI 21–27%) did not know any. In the case of diabetes, slow wound healing was commonly known as a complication (46%, CI 40–51%) as well as diabetic foot (40%, CI 37–44%). Other complications, such as uncontrolled diabetes, eye or kidney problems, and neurological or cardiovascular problems, were known by around 5–11%. Similar to hypertension, 22% (CI 18–25%) of respondents did not know any complication of diabetes.

Around half of the respondents believed everybody should be screened for hypertension and diabetes (hypertension 48%, CI 44-52%; diabetes 50%, CI 46-54%), while 43% (hypertension CI 39-47%; diabetes CI 39-46%) stated that this applies to everyone who feels sick (multiple answers possible). Only 18% (CI 14–21%) and 16% (CI 13–19%) respectively stated that people at risk should have their blood pressure and sugar checked.

In addition to general disease knowledge, we further examined attitudes and beliefs towards treatment and exposure dimensions (see Fig. 2). When asked to evaluate the statement that everybody can have hypertension, 69% (CI 66–72%) agreed, as was the case for diabetes (CI 65-72%). At the same time, 43% (CI 39–46%) agreed that having hypertension and 40% (CI 37–44%) agreed that having diabetes is destiny. Almost all respondents were afraid to have either disease (hypertension 92%, CI 90–94%; diabetes 93%, CI 91–94%), and 88% (CI 85–91%) respectively 81% (CI 78–84%) agreed that one can feel if one has hypertension respectively diabetes.

Furthermore, we found that a large majority of respondents believed that these diseases were treatable; however, more did so in the case of hypertension (91%, CI 90–93%) than in the case of diabetes (82%, CI 79–85%). Almost all respondents agreed that early treatment (95%, CI 93–97%) and regular checkups (98%, CI 97–99%) help for both diabetes and hypertension. When asked about treatment costs, 49% (CI 45–53%) thought hypertension treatment to be expensive and 62% (CI 59–66%) believed diabetes treatment to be expensive.

The factor analysis resulted in four factors which mainly capture disease-related knowledge and the notion of the disease as “serious illness” (high correlation with being afraid, ability to feel it, but also treatability; detailed results of the factor analyses displayed in Additional file 1: Table S6). As depicted in Table 1, respondents from the city Banda Aceh and with higher education had higher knowledge on hypertension and diabetes (as measured by the indices), while respondents from the lowest wealth quintile had lower index values. Respondents from the higher wealth quintiles had a stronger perception of hypertension as a severe disease (as measured with the index) compared to respondents from the lowest wealth quintile. Women, respondents with lower secondary education (compared to up to primary education), and respondents from higher wealth quintiles had a stronger perception of diabetes as a severe disease (as measured with the index). Being over 50 years old (compared to between 40 and 50) does not correlate with any of the factors.

**Table 1:** Correlates of hypertension and diabetes knowledge and beliefs

	Hypertension knowledge	Hypertension as severe disease	Diabetes knowledge	Diabetes as severe disease
**Socioeconomics**				
Age < 50	Ref	Ref	Ref	Ref
Age 50 +	0.0459	− 0.0162	− 0.0260	− 0.0240
	(− 0.0383, 0.130)	(− 0.105, 0.0723)	(− 0.116, 0.0639)	(− 0.110, 0.0623)
Male	Ref	Ref	Ref	Ref
Female	− 0.0719	0.0362	− 0.0597	0.0802**
	(− 0.160, 0.0159)	(− 0.0442, 0.117)	(− 0.140, 0.0207)	(0.00185, 0.159)
Rural	Ref	Ref	Ref	Ref
Urban	0.254***	− 0.0195	0.285***	− 0.00764
	(0.118, 0.390)	(− 0.168, 0.129)	(0.141, 0.430)	(− 0.153, 0.138)
Education				
Up to primary	Ref	Ref	Ref	Ref
Lower secondary	0.0860	− 0.115	0.0915	− 0.152**
	(− 0.0475, 0.220)	(− 0.283, 0.0522)	(− 0.0479, 0.231)	(− 0.301, − 0.00304)
Upper secondary and above	0.316***	0.00399	0.291***	− 0.0222
	(0.190, 0.443)	(− 0.134, 0.142)	(0.165, 0.417)	(− 0.149, 0.105)
Wealth				
Wealth quintile 1	Ref	Ref	Ref	Ref
Wealth quintile 2	0.206^***^	0.162	0.235^***^	0.125
	(0.0508, 0.361)	(− 0.0739, 0.398)	(0.0706, 0.400)	(− 0.104, 0.353)
Wealth quintile 3	0.164**	0.207*	0.159**	0.233**
	(0.0298, 0.298)	(− 0.0342, 0.448)	(0.0187, 0.299)	(0.00787, 0.458)
Wealth quintile 4	0.131*	0.337***	0.115*	0.362***
	(− 0.000345, 0.261)	(0.117, 0.557)	(− 0.0199, 0.251)	(0.156, 0.567)
Wealth quintile 5	0.219***	0.324***	0.245***	0.375***
	(0.0944, 0.344)	(0.105, 0.543)	(0.115, 0.375)	(0.164, 0.586)
Observations	1575	1575	1575	1575
F-stat	9.675	1.841	9.222	2.832
*p*-value	< 0.001	0.0662	< 0.001	0.00436

Regression on knowledge and belief indices created from factor analysis. 95% confidence intervals in brackets

^*^p < 0.1, ** *p* < 0.05, *** *p* < 0.01

### Screening practice

**Fig. 3:**
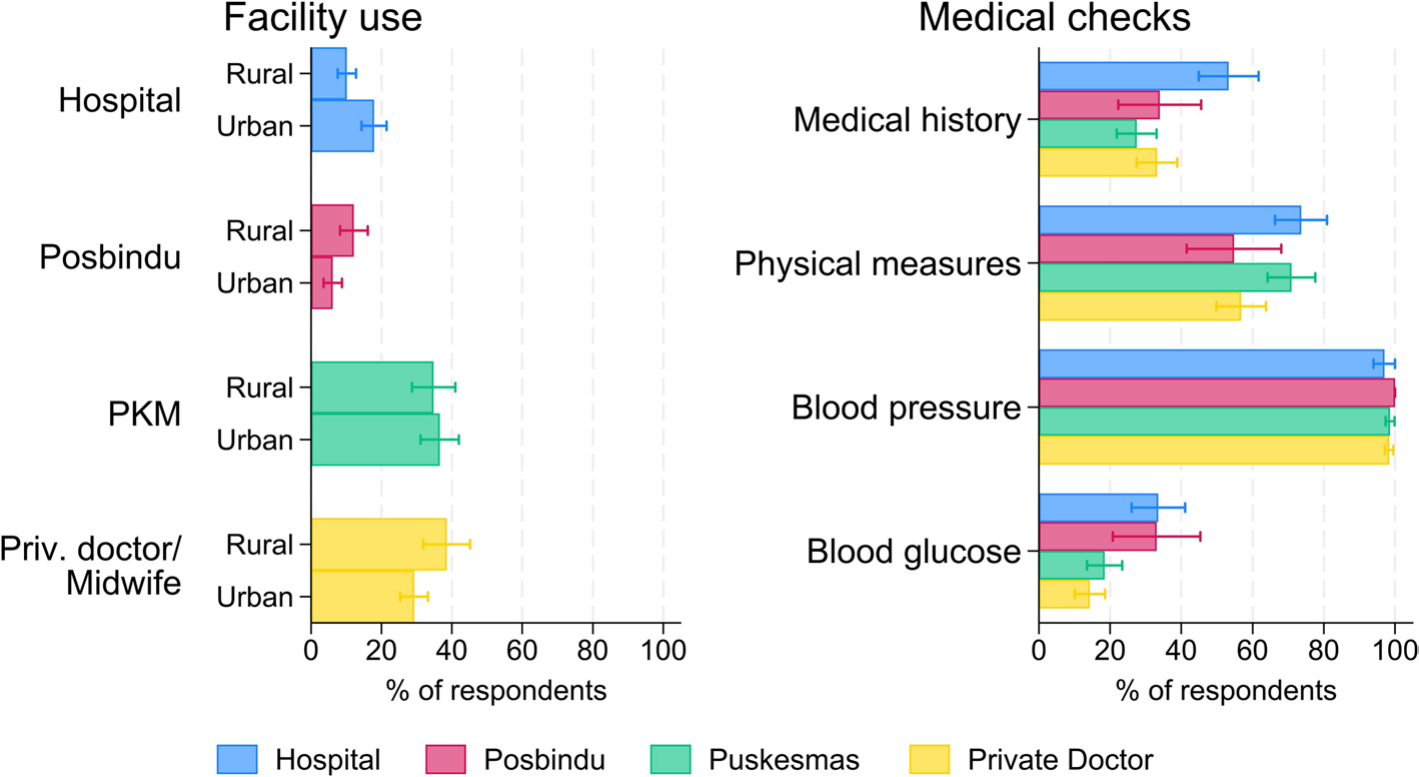
Screening practice. Note: Health care facility and conducted checks of the last screening visit. Sample restricted to respondents who reported any screening experience. Share of respondent displayed as mean estimate with 95% confidence interval

About 41% (CI 36–47%) of our respondents never had any blood pressure or blood glucose check. Among those who were screened, nearly all went in the past 5 years (Additional file 1: Fig S7). The location of the most recent check was mainly the Puskesmas or a private practice (Fig. 3). Only for 9% (CI 7–12%) of the respondents it was a Posbindu, the village-based screening service, although its usage was higher in the more rural district Aceh Besar (12%, CI: 8–16%) than in the city Banda Aceh (6%, CI 4–9%). Blood pressure was by far the most common medical check across all facilities (98%, CI 97–99%). Blood glucose was measured in only 21% (CI 17–25%) of the cases, and more often in hospitals (34%, CI 26–41%) or at Posbindu (33%, CI 21–45%) than at the Puskesmas (18%, CI 14–23%) or private practices (14%, CI 10–19%) and was often conducted in the context of other health care measures (Additional file 1: Fig S7).

Among the respondents who reported they never had a blood pressure or blood glucose check, 80% (CI 76–84%) reported that they did not go because they were not ill (Additional file 1: Fig S8). Direct or indirect costs were seldomly mentioned as reasons for not going, although about 26% (CI 21–31%) reported that they did not have the time to go.

As depicted in Table 2, wealthier individuals had higher odds to take up any screening and have had a blood pressure reading—with odds ratios (OR) ranging between 1.7 (CI 1.11–2.72) and 2.6 (CI 1.72–4.07) when compared to the lowest wealth quintile. For blood glucose, individuals age 50 and above (OR 1.5; CI 1.09–1.93), women (OR 1.5; CI 1.10–2.17), living in urban areas (OR 1.7; CI 1.08–2.75), and with higher education (lower secondary OR 1.8; CI 1.23–2.72; upper secondary and higher OR 2.0; CI 1.37–3.01) had higher odds of screening. Individuals from the lowest wealth quintile had lower odds of blood glucose screening compared to their counterparts.

**Table 2:** Correlates of screening demand: logistic regression, screening locality only for those who got screened

	**Screening uptake**			**Screening locality**		
	Ever checked	Last check incl. blood pressure	Last check incl. blood glucose	Posbindu	Puskesmas	Private
**Socioeconomics**						
Age < 50	Ref	Ref	Ref	Ref	Ref	Ref
Age 50 +	1.081	1.068	1.451**	2.037**	0.624***	0.820
	(0.847, 1.378)	(0.839, 1.359)	(1.089, 1.933)	(1.149, 3.611)	(0.472, 0.824)	(0.613, 1.098)
Male	Ref	Ref	Ref	Ref	Ref	Ref
Female	1.139	1.147	1.546**	5.531***	0.926	0.716**
	(0.882, 1.471)	(0.895, 1.468)	(1.101, 2.172)	(2.892, 10.58)	(0.677, 1.265)	(0.530, 0.967)
Rural	Ref	Ref	Ref	Ref	Ref	Ref
Urban	1.546	1.433	1.725**	0.501**	1.064	0.694*
	(0.909, 2.626)	(0.857, 2.395)	(1.080, 2.754)	(0.276, 0.908)	(0.721, 1.572)	(0.473, 1.017)
Education						
Up to primary	Ref	Ref	Ref	Ref	Ref	Ref
Lower secondary	0.766	0.791	1.831***	1.660	0.669**	1.268
	(0.555, 1.057)	(0.574, 1.092)	(1.234, 2.718)	(0.887, 3.109)	(0.451, 0.991)	(0.813, 1.975)
Upper secondary and above	0.858	0.893	2.033***	1.221	0.694**	1.169
	(0.619, 1.189)	(0.646, 1.233)	(1.374, 3.009)	(0.694, 2.146)	(0.489, 0.984)	(0.790, 1.728)
Wealth						
Wealth quintile 1	Ref	Ref	Ref	Ref	Ref	Ref
Wealth quintile 2	1.737**	1.711**	0.583*	1.349	1.425	0.909
	(1.111, 2.716)	(1.096, 2.670)	(0.326, 1.042)	(0.651, 2.794)	(0.861, 2.360)	(0.534, 1.548)
Wealth quintile 3	2.230***	2.148***	0.425***	0.578	1.133	1.062
	(1.490, 3.337)	(1.423, 3.241)	(0.253, 0.715)	(0.249, 1.340)	(0.714, 1.798)	(0.612, 1.843)
Wealth quintile 4	2.646***	2.609***	0.575*	0.724	0.829	1.585*
	(1.720, 4.070)	(1.703, 3.997)	(0.298, 1.111)	(0.306, 1.717)	(0.494, 1.392)	(0.920, 2.733)
Wealth quintile 5	2.327***	2.120***	0.592*	0.527	0.487***	1.602*
	(1.489, 3.637)	(1.356, 3.314)	(0.335, 1.046)	(0.225, 1.233)	(0.300, 0.790)	(0.929, 2.763)
Observations	1575	1575	1575	950	950	950
F-stat	2.725	2.641	5.001	4.901	4.696	2.148
*p*-value	0.00591	0.00751	< 0.001	< 0.001	< 0.001	0.0297

Adjusted odds ratios. Regressions on the screening locality are restricted to respondents reporting any screening. 95% confidence intervals in brackets

^*^p < 0.1, ** *p* < 0.05, *** *p* < 0.01

To further investigate where the last blood pressure or blood glucose check was conducted, we focused on Posbindu, Puskesmas, and private doctors. Respondents above age 50 had twice the odds (OR 2.0; CI 1.15–3.61) to visit a Posbindu and lower odds (OR 0.62; CI 0.47–0.82) to visit a Puskesmas compared to younger respondents. Women had about five times higher odds than men to visit Posbindu as their last screening location (OR 5.5; CI 2.89–10.58), while they had lower odds than men to mention a private doctor as last location (OR 0.7; CI 0.53–0.97). Respondents from the city of Banda Aceh had lower odds than respondents from the rural district Aceh Besar to mention Posbindu (OR 0.5; CI 0.28–0.91) or a private facility (OR 0.7; CI 0.47–1.02) as last screening location. Respondents with no or primary education had higher odds to visit a Puskesmas compared to respondents with higher education levels (lower secondary OR 0.7; CI 0.45–0.99; upper secondary and higher OR 0.7; CI 0.49–0.98). When including the knowledge and belief indices obtained from the factor analyses above, the results stayed largely the same, with education gradients becoming significant for ever being checked and being checked for blood pressure, and the odds ratios for the fourth and fifth wealth quintile becoming insignificant for blood glucose checks and checks at private practices (Additional file 1: Table S7). The knowledge indices were positively correlated with screening uptake, and the notion of diabetes as a serious illness was negatively correlated with glucose checks.

## Discussion

In a random sample of 2080 adults that were indicated for diabetes and hypertension screening according to WHO PEN and national guidelines, we find that CVD knowledge and beliefs, disease- and provider-specific challenges, and socioeconomic gradients constitute barriers to screening uptake in Aceh, Indonesia. Our findings contribute to an understanding of how to increase diabetes and hypertension care uptake in Indonesia, the fourth most populous country in the world and among those with the highest CVD death rates in Southeast Asia.

In Aceh, common financial and time barriers to diabetes and hypertension screening uptake are largely reduced through free, local screening offers. Especially with the village-based screening program, it is not necessary to organize or pay for traveling, and screening takes place in a familiar setting. Nevertheless, we find that knowledge and belief barriers remain. Respondents are generally aware of diabetes and hypertension, with high knowledge of severe complications, the role of diet as a risk factor, and practices to control the conditions. However, a majority is unaware of the potentially asymptomatic nature of these conditions, of who should seek screening, and of key lifestyle risk factors. In line with this, we find high consumption of tobacco and SSB, low physical activity, and large shares of respondents that have never taken up screening before. At the same time, we find that the socioeconomic characteristics of urban location, higher education, and wealth are associated with greater diabetes- and hypertension-related knowledge. These patterns closely mirror findings from other studies in Southeast Asian, middle-income countries, where in-depth knowledge and screening uptake for diabetes and hypertension remain similarly low and underlie comparable socioeconomic gradients [10, 14, 15]. As such, making screening guidelines and general disease-related knowledge more salient to the at-risk population, and especially to those with lower socioeconomic status, may be key target areas for increasing CVD care uptake in this setting.

A major advantage of our study is the direct comparability of hypertension and diabetes to uncover disease-specific barriers to screening uptake. While awareness of and attitudes towards these two conditions are similar, we find substantial differences between hypertension and diabetes screening practices. When asked about the last screening visit, nearly all respondents state that blood pressure was measured, while blood glucose was measured in less than every fourth case. These patterns could reflect a more targeted effort by the health system to conduct screenings for hypertension as the more prevalent condition in comparison to diabetes. However, they may also point to several disease-specific barriers to screening. First, diabetes screening may underlie additional barriers due to its more resource-intensive and invasive measurement mode. This is in line with findings from recent studies on up to 57 LMICs, including Indonesia, that show diabetes screening to be less prevalent than hypertension screening among the general population, among individuals meeting screening criteria, and among individuals with diabetes and hypertension respectively [28–30]. Second, the reported blood pressure measurements may have occurred in the context of diagnosing other health conditions, e.g., kidney disease, preeclampsia, or sepsis, and as such may not reflect dedicated hypertension screening. This is supported by literature showing that high rates of blood pressure measurements do not translate into similarly high hypertension diagnosis rates [30].

Adding further nuance to this, we show that while blood pressure measurements were reported at all facility types, blood glucose tests were mainly conducted in hospitals and Posbindu—potentially suggesting that facility choice may be related to the type of screening visitors receive. Furthermore, we show that both the facility choice and type of screening underlie complex socioeconomic gradients. For instance, we find that older respondents and women are more likely to visit Posbindu than younger and male respondents. While Posbindu aims to serve the general population, qualitative studies find that it is more heavily used by older and female visitors, hinting towards a perceived labeling of Posbindu as a program for the elderly [31], and thereby potentially contributing to socioeconomic gradients in who is receiving blood glucose measurements [8, 9]. These findings shed light on two potential routes to increase diabetes and hypertension screening rates. On the one hand, a greater awareness of the Posbindu program and its target population could channel the population at risk towards dedicated screening services. However, major bottlenecks might remain as Posbindu regularly lacks sufficient resources to offer a comprehensive CVD screening with measurements beyond blood pressure and blood glucose [9]. On the other hand, opportunistic screening at other facilities might be expanded, for example, with point-of-care machines, which decrease the expense of opportunistic screening. A study based on other middle-income countries showed that between 12 and 37% of hypertensive patients visited a health care provider in the previous month [32], demonstrating the potential of covering a large share of the population at risk. Moreover, opportunistic screening might reduce socioeconomic gaps in CVD screening, as in Indonesia, socioeconomic differences tend to be smaller in primary care utilization than in screening utilization [33].

Our study comes with some limitations. We measured screening behavior based on respondents’ self-reports, which might introduce recall and other reporting biases. However, as medical examinations are not part of our respondents’ everyday life and thus might be easier to remember, and we do not inform them upfront which screenings are recommended and at which frequency, we deem the potential bias to be low. Also, we assessed disease knowledge with open-ended questions. While this allows us to measure the facts respondents are able to recall themselves unaided, it does not necessarily measure disease recognition and labeling, as for example in the case of vignettes [34].

## Conclusions

Diabetes and hypertension constitute major global health burdens, and Indonesia is lagging behind in addressing unmet care needs compared to other middle-income countries [32]. Improving CVD care is expected to lead to substantial gains in life expectancy: Controlled blood pressure is associated with a gain of 5 years in life expectancy for men, and 6 years for women in Indonesia [35]. This analysis offers important insights into how to advance towards these gains by identifying inadequate CVD knowledge, disease- and provider-specific challenges, and socioeconomic gradients as key barriers to diabetes and hypertension screening uptake.

## Supplementary Information

Additional file 1: Native language abstract, details on the variable construction and additional analyses. Table S1: Variable definitions for screening indication and risk factors. Table S2: Belief statements. Table S3: Comparison of sample characteristics with SUSENAS 2019. Table S4: Number and percent of missingness in all variables of interest. Table S5: Correlates of risk with knowledge. Table S6: Factor analyses. Table S7: Regression for screening uptake and screening locality including knowledge and belief indices. Fig S1. Reported health complaints. Fig S2. Smoking risk in survey sample. Fig S3. Smoking risk SUSENAS 2019 in study region compared to Aceh province and Indonesia. Fig S4. Kind and number of sugared drinks consumed by respondent on a normal day. Fig S5. Days with any physical activity or walking (more than 10 minues continuously) during the past week. Fig S6. Duration and percent of respondents doing physical activity and walk on an active day during the last week. Fig S7. Details on prior screening visit. Fig S8. Reasons never being screened.

## Data Availability

The dataset(s) supporting the conclusions of this article are available in the Göttingen Research Online repository, 10.25625/R7MQJ1, at https://data.goettingen-research-online.de [36]. The questionnaire underlying this study is publicly available in the repository.
