# Supplementary material for: Hypertension and diabetes screening uptake in adults aged 40–70 in Indonesia: a knowledge, attitudes, and practices study
Source: BMC Glob Public Health. 2025 May 13;3:44. doi: 10.1186/s44263-025-00157-7 (PMC12076961; doi:10.1186/s44263-025-00157-7)
Supplement: Supplementary file 1 — Additional file 1: Native language abstract, details on the variable construction and additional analyses. Table S1: Variable definitions for screening indication and risk factors. Table S2: Belief statements. Table S3: Comparison of sample characteristics with SUSENAS 2019. Table S4: Number and percent of missingness in all variables of interest. Table S5: Correlates of risk with knowledge. Table S6: Factor analyses. Table S7: Regression for screening uptake and screening locality including knowledge and belief indices. Fig S1. Reported health complaints. Fig S2. Smoking risk in survey sample. Fig S3. Smoking risk SUSENAS 2019 in study region compared to Aceh province and Indonesia. Fig S4. Kind and number of sugared drinks consumed by respondent on a normal day. Fig S5. Days with any physical activity or walking (more than 10 minues continuously) during the past week. Fig S6. Duration and percent of respondents doing physical activity and walk on an active day during the last week. Fig S7. Details on prior screening visit. Fig S8. Reasons never being screened. [file 44263_2025_157_MOESM1_ESM.pdf]

Additional file 1

Native language abstract, details on the variable construction, and additional analyses

Additional File 1: Native Language Abstract

This translation in Bahasa Indonesia was submitted by the authors and we reproduce it as supplied. It has not been peer reviewed. Our editorial processes have only been applied to the original abstract in English, which should serve as reference for this article. This translated abstract is published under the same license as the article.

Latar Belakang: Diabetes dan hipertensi merupakan krisis kesehatan global utama. Namun, Indonesia masih menghadapi tantangan dalam mencapai hasil perawatan yang optimal dibandingkan dengan negara-negara berpendapatan menengah lainnya. Penelitian ini mengeksplorasi hambatan dalam pelaksanaan skrining sebagai langkah awal untuk mendapatkan perawatan, khususnya pada orang dewasa berusia 40-70 tahun di Aceh, Indonesia.

Metode: Kami menganalisis data individu terkait skrining diabetes dan hipertensi di Banda Aceh dan Aceh Besar pada tahun 2019-2020. Melalui pengambilan sampel acak dua tahap, kami mengumpulkan data survei dari 2.080 orang dewasa yang memiliki indikasi skrining namun belum pernah menjalani pemeriksaan sesuai pedoman Paket Intervensi Esensial Penyakit Tidak Menular dari Organisasi Kesehatan Dunia. Data disesuaikan untuk desain survei kompleks guna mengevaluasi: (1) proporsi responden dengan indikasi skrining dan faktor risiko, (2) tingkat pengetahuan, sikap, dan praktik terkait penyakit, serta (3) hubungan antara skrining dengan karakteristik sosial ekonomi, pengetahuan, dan sikap menggunakan regresi linier dan logistik multivariat.

Hasil: Meskipun sebagian besar responden mengetahui diabetes dan hipertensi, mayoritas tidak memahami faktor risiko utama, sifat penyakit yang dapat bersifat asimtomatik, serta pentingnya skrining. Sekitar 41% responden belum pernah melakukan pemeriksaan tekanan darah atau glukosa, dengan alasan utama “tidak merasa sakit”. Pemeriksaan glukosa darah sangat jarang dilakukan. Lokasi pedesaan dan tingkat pendidikan rendah berasosiasi dengan pengetahuan penyakit yang lebih rendah, sementara tingkat kekayaan yang lebih rendah terkait dengan pengetahuan yang terbatas dan rendahnya penerimaan terhadap skrining.

Kesimpulan: Hambatan terhadap skrining di Aceh mencakup kesalahpahaman terkait diabetes dan hipertensi, keterbatasan penyedia layanan, khususnya dalam pelaksanaan tes glukosa darah, serta ketimpangan sosial ekonomi.

Table S1: Variable definitions for screening indication and risk factors

WHO PEN risk factors	Variable definition in the study
Diabetes screening	
From the age of 40 onwards	Age>=40 years (sample inclusion criteria)
Overweight or obese	Not available
Physical inactivity	Engaging in less than 120 minutes of continuous physical activity or walking within the past seven days
First degree relative with diabetes	Household member with diabetes
History of CVD, hypertension, or dyslipidemia	History of heart attack/stroke or high cholesterol (hypertension not applicable based on sample exclusion criteria)
History of gestational diabetes or preeclampsia	Not available
Hypertension screening (including blood pressure screening within CVD detection)	
From the age of 40 onwards	Age>= 40 years (sample inclusion criteria)
Overweight or obese	Not available
Smoking/former smoking	Smoking
History of heart attack/stroke	History of heart attack/stroke
History of Hypertension/diabetes	Not applicable (sample exclusion criteria)
First-degree relative with premature CVD, diabetes or kidney disease	Household member with a history of heart attack/stroke or diabetes
**Additional reported risk factors (not part of indices)**	
Any consumption of sugar-sweetened beverages	
History of Inflammatory arthritis	

All items are used individually and as composite indices for diabetes screening and for hypertension screening to describe the screening indication and risk factors in the sample.

Table S2: Belief statements

In principle, everyone has the chance of experiencing [hypertension/diabetes]
[Hypertension/diabetes] is treatable
It's expensive to have [hypertension/diabetes]
I am afraid to have [hypertension/diabetes]
There is nothing one can do to prevent [hypertension/diabetes], it is destiny
One can feel whether you have [hypertension/diabetes]
Checking your blood pressure regularly helps to detect [hypertension/diabetes] early
It makes a difference to start treatment early

Each of the statements were asked separately for hypertension and diabetes. Respondents could answer on a 4-point Likert scale, ranging from "strongly agree" to "strongly disagree". The categories "strongly agree" and "agree" as well as "disagree" and "strongly disagree" were aggregated for the analyses.

Table S3: Comparison of sample characteristics with SUSENAS 2019

	SUSENAS Banda Aceh, Aceh Besar	Survey sample
Age	49.3712	50.1478**
	(0.2783)	(0.1799)
Female	0.5785	0.6411***
	(0.0187)	(0.0105)
Education		
- Up to primary	0.2340	0.2987***
	(0.0161)	(0.0099)
- Lower secondary	0.1656	0.2203***
	(0.0142)	(0.0091)
- Upper secondary and above	0.5916	0.4810***
	(0.0188)	(0.0107)
Banda Aceh	0.4634	0.4300
	(0.0166)	(0.0061)
N	950	2,079

Standard errors accounting for survey design (sampling weights in SUSENAS, district stratification in both samples) below mean; stars indicate significant difference from mean listed in previous column based on adjusted Wald test, * 0.1 ** 0.05 *** 0.01; the SUSENAS sample is also restricted to 40-70 year olds who have access to a mobile phone to match the target population as closely as possible.

Table S4: Number and percent of missingness in all variables of interest

	Observations included in analysis	Number Missings	% missing
**Socioeconomic Characteristics**			
Age 50+	2,078	2	0.10
Female	2,077	3	0.14
Education	2,079	1	0.05
Wealth quintile	2,079	1	0.05
Area	2,080	0	0.00
Risk indicators			
Current smoker	2,076	4	0.19
Low physical activity	2,077	3	0.14
Stroke/heart attack	2,029	51	2.45
Cholesterol	2,029	51	2.45
Household with stroke/heart attack	2,064	16	0.77
Household with diabetes	2,064	16	0.77
Any sugar-sweetened beverages	2,079	1	0.05
Inflammatory arthritis	2,028	52	2.50
**Hypertension knowledge**			
Risk factors	2,057	23	1.11
Complications	2,057	23	1.11
Control	2,057	23	1.11
Screening target	2,080	0	0.00
Everyone can have it	1,972	108	5.19
It's treatable	2,002	78	3.75
Treatment is expensive	1,913	167	8.03
I'm afraid to have it	2,051	29	1.39
Regular checks help	2,031	49	2.36
Early treatment helps	2,030	50	2.40
Likely to have it	2,002	78	3.75
It's destiny	2,022	58	2.79
You can feel it	1,999	81	3.89
**Diabetes knowledge**			
Risk factors	2,008	72	3.46
Complications	2,007	73	3.51
Control	2,007	73	3.51
Screening target	2,080	0	0.00
Everyone can have it	1,929	151	7.26
It's treatable	1,940	140	6.73
Treatment is expensive	1,869	211	10.14
I'm afraid to have it	2,000	80	3.85
Regular checks help	1,972	108	5.19
Early treatment helps	1,976	104	5.00
Likely to have it	1,942	138	6.63
It's destiny	1,968	112	5.38
You can feel it	1,851	229	11.01
**Screening practices**			
Ever had blood pressure or blood glucose checked	2,071	9	0.43
Last check with blood pressure	2,071	9	0.43
Last check with blood glucose	2,071	9	0.43
**Last screening visit**			
Hospital	1,220	1	0.05
Posbindu	1,220	1	0.05
Puskesmas	1,220	1	0.05
Private doctor/midwive	1,220	1	0.05
Other	1,220	1	0.05
Medical history	1,220	1	0.05
Physical measures	1,220	1	0.05
Blood pressure	1,220	1	0.05
Blood glucose	1,220	1	0.05

The missingness indicated in this table includes item refusal and stating ‘don’t know’. The full sample for the last screening visit is lower as only respondents who stated to have ever had a BP or BG screening are included.

Table S5: Correlates of risk with knowledge

	Hypertension				Diabetes			
	Smoking	Sed. life style	Dietary factors	Genetic	Smoking	Sed. life style	Dietary factors	Genetic
	Odds ratios							
Current smoker	2.573***<br>(0.578)				1.232<br>(0.565)			
Low physical activity		1.236<br>(0.195)				1.349<br>(0.274)		
Any SSB			0.814<br>(0.114)				0.895<br>(0.143)	
Household preposition				1.497**<br>(0.262)				1.359**<br>(0.183)
	Adjusted odds ratios							
Current smoker	1.636<br>(0.554)				2.546<br>(1.928)			
Low physical activity		1.124<br>(0.191)				1.228<br>(0.269)		
Any SSB			0.835<br>(0.122)				0.961<br>(0.156)	
Household preposition				1.459**<br>(0.264)				1.314*<br>(0.190)
Observations	2050	2051	2053	2038	2002	2003	2005	1992

Odds ratios and adjusted odds ratio for knowing the different risk factors, controlling for age, sex, education and location. Household preposition is a binary variable equal to one if any household member has diabetes or ever had a heart attack or stroke. Standard errors in parentheses. * $p < 0.1$, ** $p < 0.05$, *** $p < 0.01$

Table S6: Factor analyses

	HT Knowledge	HT Serious illness	DM Knowledge	DM Serious illness
Eigenvalue	1.55	1.45	1.79	1.32
Variance of rotated factor	1.52	1.48	1.77	1.34
Knows any risk factor	0.63	0.01	0.65	0.03
Knows any complication	0.58	0.00	0.63	-0.01
Knows any control measure	0.67	0.03	0.72	0.03
Knows anyone affected	0.23	-0.00	0.33	0.00
Everyone should be screened	-0.09	0.16	-0.12	0.18
Everyone can have it	0.32	-0.17	0.28	-0.22
It's treatable	0.06	-0.04	-0.03	-0.05
Treatment is expensive	0.05	-0.17	0.21	-0.17
I'm afraid to have it	0.02	0.66	0.04	0.67
It's destiny	-0.14	0.07	-0.10	0.10
You can feel it	0.12	0.62	0.15	0.46
Regular checks help	0.08	0.04	0.06	0.07
Early treatment helps	-0.07	0.75	-0.05	0.71
Likely to have it	0.35	-0.14	0.38	-0.21

Factor eigenvalues, variances of rotated factors, and matrix of rotated factor loadings, Factors were rotated using oblique oblimin rotation. Only factors with eigenvalue 1 or above were retained.

Table S7: Regression for screening uptake and screening locality including knowledge and belief indices

	**Screening Uptake**			**Screening Locality**		
	Ever checked	Last check incl. blood pressure	Last check incl. blood glucose	Posbindu	Puskesmas	Private
Socioeconomics						
Age 50+	1.071	1.041	1.466***	1.886**	0.631***	0.834
	[0.837,1.371]	[0.818,1.324]	[1.102,1.951]	[1.055,3.368]	[0.476,0.835]	[0.622,1.118]
Female	1.202	1.208	1.703***	5.259***	0.976	0.730**
	[0.927,1.559]	[0.939,1.553]	[1.196,2.424]	[2.734,10.12]	[0.715,1.332]	[0.537,0.992]
Urban	1.334	1.250	1.609**	0.581*	0.987	0.683**
	[0.785,2.265]	[0.748,2.088]	[1.017,2.545]	[0.312,1.085]	[0.656,1.485]	[0.470,0.995]
Education						
Lower secondary	0.704**	0.735*	1.603**	1.387	0.689*	1.320
	[0.500,0.990]	[0.523,1.034]	[1.035,2.481]	[0.753,2.556]	[0.464,1.022]	[0.842,2.069]
Upper secondary	0.701**	0.733*	1.818***	1.090	0.672**	1.193
and above	[0.494,0.994]	[0.521,1.032]	[1.206,2.740]	[0.605,1.964]	[0.473,0.956]	[0.798,1.784]
Wealth						
Wealth quintile 2	1.592**	1.588**	0.577*	1.776	1.272	0.870
	[1.016,2.493]	[1.012,2.493]	[0.332,1.002]	[0.751,4.199]	[0.750,2.157]	[0.514,1.471]
Wealth quintile 3	2.163***	2.074***	0.479***	0.794	1.045	1.011
	[1.412,3.313]	[1.340,3.210]	[0.295,0.778]	[0.339,1.863]	[0.659,1.657]	[0.577,1.771]
Wealth quintile 4	2.666***	2.607***	0.730	1.144	0.740	1.449
	[1.684,4.220]	[1.657,4.101]	[0.378,1.411]	[0.460,2.843]	[0.443,1.235]	[0.839,2.501]
Wealth quintile 5	2.182***	1.985***	0.722	0.877	0.425***	1.473
	[1.364,3.489]	[1.251,3.150]	[0.398,1.309]	[0.356,2.159]	[0.257,0.703]	[0.848,2.560]
Indices						
Hypertension	1.412***	1.802***		1.094	1.102	1.011
knowledge	[1.170,1.703]	[1.490,2.179]		[0.698,1.715]	[0.865,1.404]	[0.735,1.390]
Hypertension	1.026	0.942		0.584***	1.528**	1.286
serious illness	[0.832,1.265]	[0.812,1.093]		[0.389,0.876]	[1.071,2.181]	[0.890,1.857]
Diabetes	1.292**		1.632***	0.867	1.125	0.969
knowledge	[1.063,1.569]		[1.135,2.348]	[0.575,1.308]	[0.865,1.463]	[0.743,1.265]
Diabetes serious	0.901		0.592***	1.033	0.816	0.937
illness	[0.710,1.142]		[0.502,0.697]	[0.660,1.616]	[0.561,1.188]	[0.638,1.375]
Obs.	1575	1575	1575	950	950	950
F-stat	4.114	5.045	10.93	5.440	3.848	1.621
p-value	<0.001	<0.001	<0.001	<0.001	<0.001	0.0872

Adjusted odds ratios. Reference categories are: Age below 50 years, being male, rural, up to primary education, and wealth quintile 1. Regressions on the screening locality are restricted to respondents reporting any screening. 95% confidence intervals in brackets. * $p < 0.1$, ** $p < 0.05$, *** $p < 0.01$

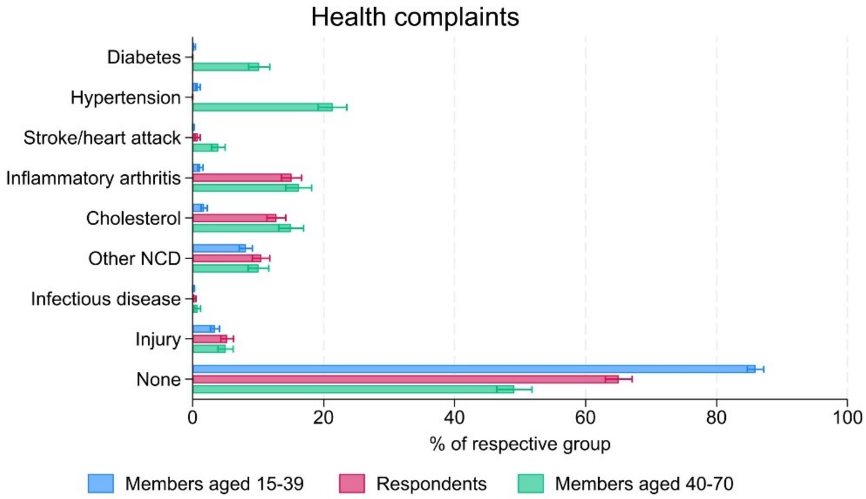

Fig S1 Reported health complaints

Health complaints of each member are reported by the main respondent. By design, respondents did not have a prior diabetes or hypertension diagnosis. Displayed shares in each group as mean estimate with 95% confidence interval.

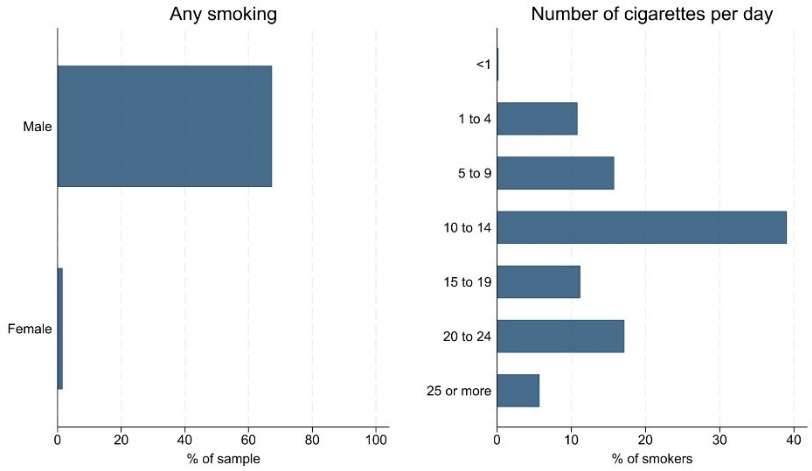

Fig S2 Smoking risk in survey sample

Bars depict the share of respondents who stated to currently smoke at the time of the survey (left) and the usual number of cigarettes per day (right).

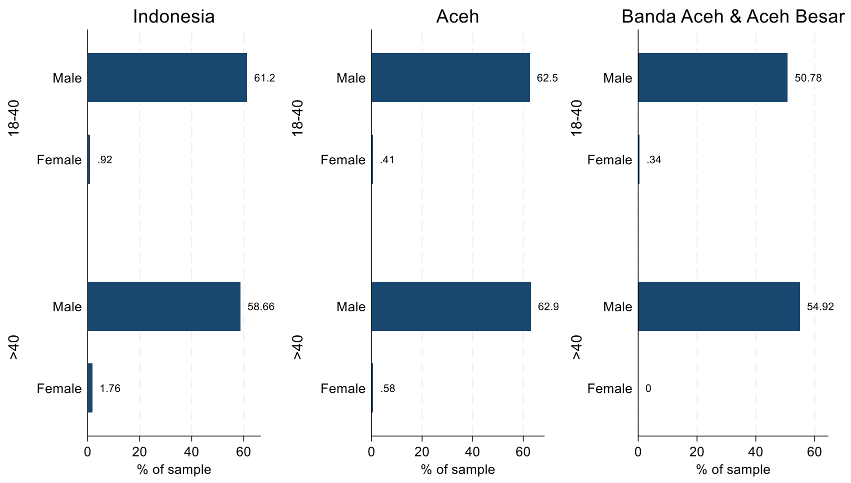

Fig S3 Smoking risk SUSENAS 2019 in study region compared to Aceh province and Indonesia
Bars depict share of the respective gender and age group who was smoking during the month prior to the survey.

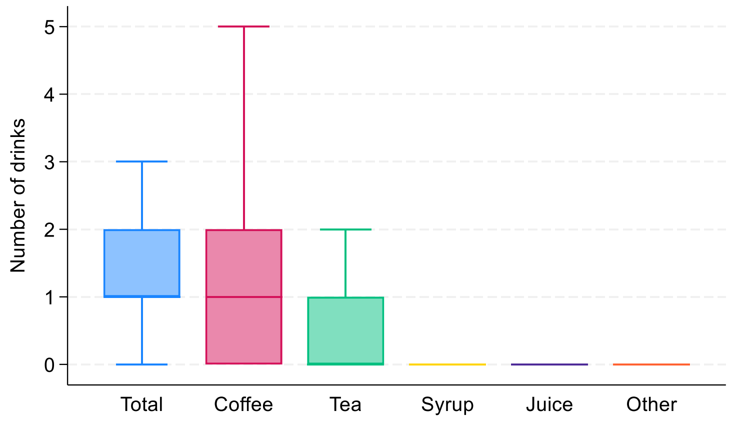

Fig S4 Kind and number of sugared drinks consumed by respondent on a normal day
Boxplot excludes outliers.

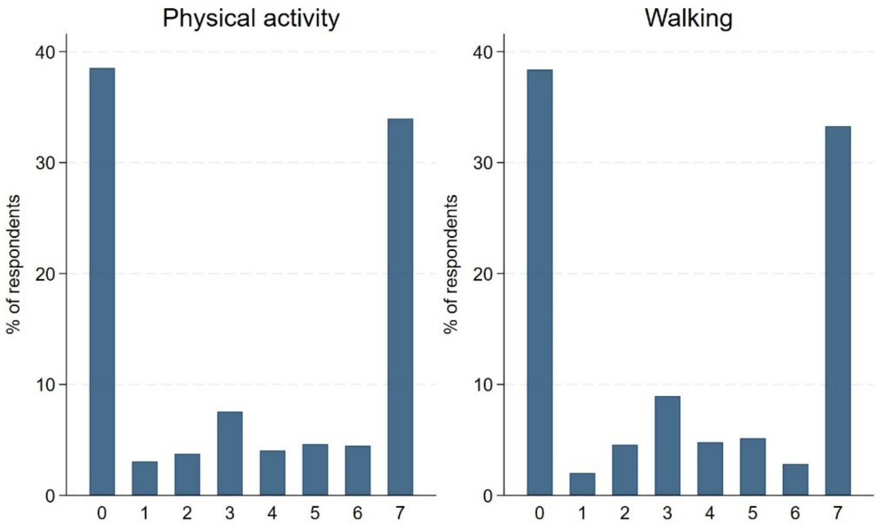

Fig S5. Days with any physical activity or walking (more than 10 minues continuously) during the past week
Bars depict the share of respondents reporting the respective number of days of physical activity (left) and walking (right) during the past week.

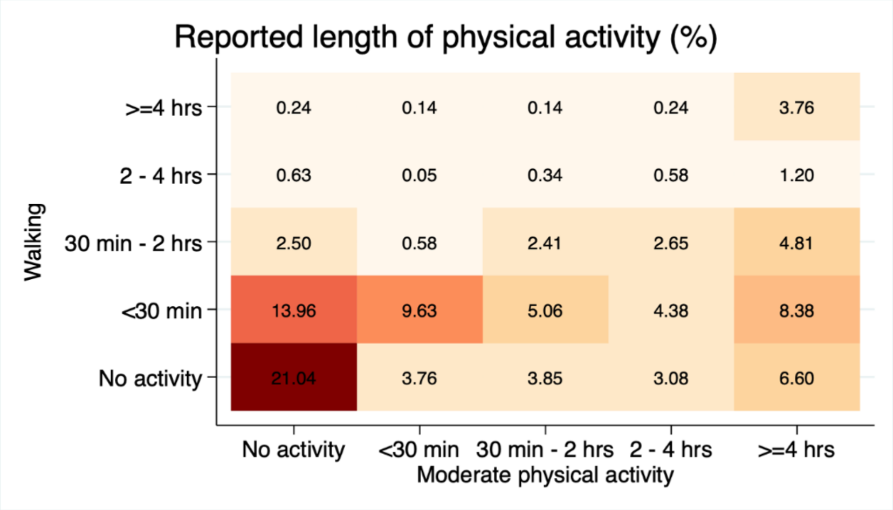

Fig S6 Duration and percent of respondents doing physical activity and walk on an active day during the last week
Each field reports the percentage of respondents with the respective combination of walking and physical activity in the past week. Fields are colored according to the percentage number, with darker field representing higher numbers.

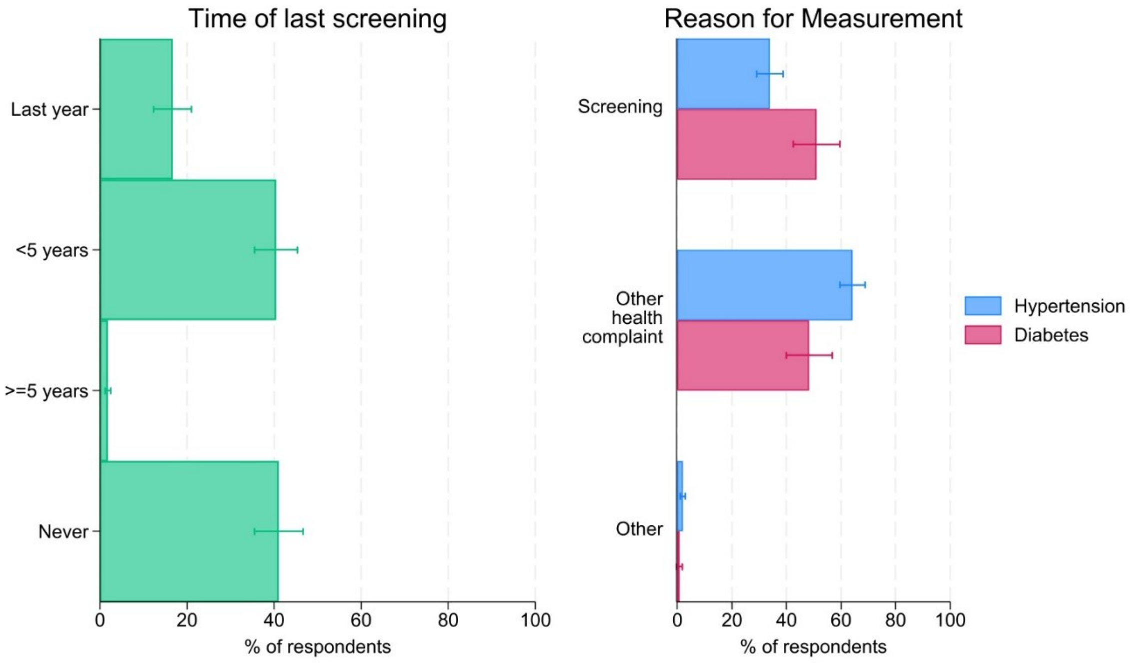

Fig S7. Details on prior screening visit

Sample restricted to respondents who reported any prior screening experience. Shares displayed as mean estimate with 95% confidence intervals.

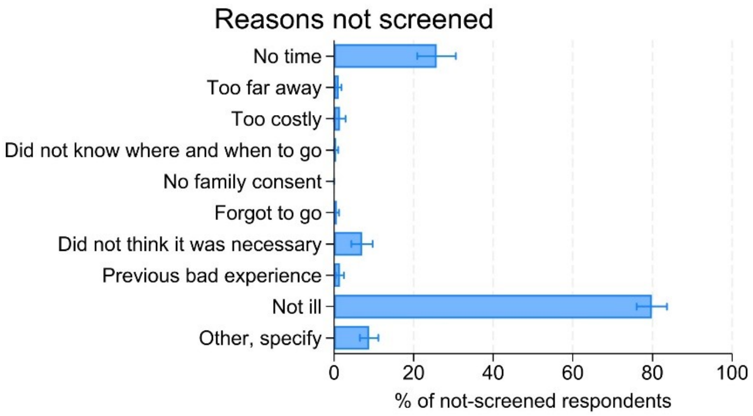

Fig S8. Reasons never being screened
Sample restricted to respondents who did not report any prior screening experience. Shares displayed as mean estimate with 95% confidence intervals.

STROBE Checklist

	Item No	Recommendation
**Title and abstract**	1	(*a*) Indicate the study's design with a commonly used term in the title or the abstract. **The title includes this information**
		(*b*) Provide in the abstract an informative and balanced summary of what was done and what was found. **This information is included in the abstract.**
**Introduction**		
Background/rationale	2	Explain the scientific background and rationale for the investigation being reported. **This information is contained throughout the introduction**
Objectives	3	State specific objectives, including any prespecified hypotheses. **This information is provided in the final paragraph of the introduction.**
**Methods**		
Study design	4	Present key elements of study design early in the paper. **This information is provided in the Methods section, in the subsection on Data Sources and Variable Definitions and Measurements**
Setting	5	Describe the setting, locations, and relevant dates, including periods of recruitment, exposure, follow-up, and data collection. **This information is provided in the Methods, in the subsection on Data Sources.**
Participants	6	(*a*) *Cross-sectional study*—Give the eligibility criteria, and the sources and methods of selection of participants. **This information is provided in the Methods, in the subsection on Data Sources.**
		(*b*) *Case-control study*—For matched studies, give matching criteria and the number of controls per case. **We did not use a matched design.**
Variables	7	Clearly define all outcomes, exposures, predictors, potential confounders, and effect modifiers. Give diagnostic criteria, if applicable. **Please see Methods, subsections on Variable Definitions and Measurements and Statistical Analyses.**
Data sources/ measurement	8*	For each variable of interest, give sources of data and details of methods of assessment (measurement). Describe comparability of assessment methods if there is more than one group. **Please see Methods, subsections on Variable Definitions and Measurements and Statistical Analyses.**
Bias	9	Describe any efforts to address potential sources of bias. **Please see Methods, subsections on Variable Definitions and Measurements and Statistical Analyses, and the Discussion.**
Study size	10	Explain how the study size was arrived at. **This information can be found in Methods, under the section on Data Sources.**
Quantitative variables	11	Explain how quantitative variables were handled in the analyses. If applicable, describe which groupings were chosen and why. **Please see the Methods section for this information, under the subheadings Data Sources, Variable Definitions and Measurements and Statistical Analyses.**
Statistical methods	12	(*a*) Describe all statistical methods, including those used to control for confounding. **Please see the Methods section, in the subsection Statistical Analyses.**
		(*b*) Describe any methods used to examine subgroups and interactions. **Please see the Methods section, in the subsection Statistical Analyses.**
		(*c*) Explain how missing data were addressed. **This information can be found in Methods, in the subsection Statistical Analyses.**
		(*d*) If applicable, describe analytical methods taking account of sampling strategy. **Please see the Methods section, in the subsection Statistical Analyses.**
		(*e*) Describe any sensitivity analyses. **Please see the Methods section, in the subsection Statistical Analyses, and Results.**

Results		
Participants	13*	(a) Report numbers of individuals at each stage of study—eg numbers potentially eligible, examined for eligibility, confirmed eligible, included in the study, completing follow-up, and analysed. **This information can be found in Methods, under the section on Data Sources.**
		(b) Give reasons for non-participation at each stage. **This information can be found in Methods, under the section on Data Sources.**
		(c) Consider use of a flow diagram
Descriptive data	14*	(a) Give characteristics of study participants (eg demographic, clinical, social) and information on exposures and potential confounders. **This information can be found in the Results section, specifically in the Screening Indication subsection and the Additional file 1: Table S3**
		(b) Indicate number of participants with missing data for each variable of interest **This information can be found in the Results section, specifically in the Screening Indication subsection and the Additional file 1: Table S4**
Outcome data	15*	Report numbers of outcome events or summary measures. **This information can be found in the Results section, Figure 1 -3.**
Main results	16	(*a*) Give unadjusted estimates and, if applicable, confounder-adjusted estimates and their precision (eg, 95% confidence interval). Make clear which confounders were adjusted for and why they were included. **This information can be found in the Results section, Figure 1-3, Table 1-2, and in the Additional file 1: Table S5 and S7.**
		(*b*) Report category boundaries when continuous variables were categorized. **This can be found in the Results section, Table 1-2.**
		(*c*) If relevant, consider translating estimates of relative risk into absolute risk for a meaningful time period. **This is not applicable to this study.**
Other analyses	17	Report other analyses done—eg analyses of subgroups and interactions, and sensitivity analyses. **These results are reported in the results section, and the Additional file 1.**
**Discussion**		
Key results	18	Summarise key results with reference to study objectives. **This is presented in paragraphs 1-4 of the Discussion section.**
Limitations	19	Discuss limitations of the study, taking into account sources of potential bias or imprecision. Discuss both direction and magnitude of any potential bias. **This is presented in paragraph 5 of the Discussion section.**
Interpretation	20	Give a cautious overall interpretation of results considering objectives, limitations, multiplicity of analyses, results from similar studies, and other relevant evidence. **This is presented in paragraphs 1-5 of the Discussion section.**
Generalisability	21	Discuss the generalisability (external validity) of the study results. **This is presented in paragraphs 2-4 of the Discussion section.**
**Other information**		
Funding	22	Give the source of funding and the role of the funders for the present study and, if applicable, for the original study on which the present article is based. **We have provided this information in the section entitled "Funding" following the main manuscript.**
